# Targeting COVID-19 vaccine hesitancy among nurses in Shanghai: A latent profile analysis

**DOI:** 10.3389/fpubh.2022.953850

**Published:** 2022-09-14

**Authors:** Enming Zhang, Zhengyue Dai, Caifeng Wang, Jiale Hu, Suxing Wang, Lin Zhang, Qiong Fang

**Affiliations:** ^1^School of Nursing, Shanghai Jiao Tong University School of Medicine, Shanghai, China; ^2^College of Health Professions, Virginia Commonwealth University, Richmond, VA, United States; ^3^School of Public Health, Shanghai Jiao Tong University School of Medicine, Shanghai, China

**Keywords:** COVID-19, COVID-19 vaccine, vaccine hesitancy, nurses, latent profile analysis

## Abstract

**Objectives:**

This study aims to clarify the profiles of the psychological antecedents of vaccine hesitancy among Shanghai nurses with a person-centered approach.

**Methods:**

A population-based cross-sectional online survey was conducted on Shanghai nurses from July to August 2021 (*N* = 1,928). In the online survey, participants were asked to report their sociodemographic, the 5C vaccine hesitancy components, their knowledge level of COVID-19 vaccine and vaccination, and the COVID-19 vaccination uptake intention and attention to vaccine news. Latent profile analysis was used to reveal distinct profiles of vaccine hesitancy.

**Results:**

The results revealed four profiles, including “believers” (68.9%; high confidence and collective responsibility), “free riders” (12.7%; similar characteristics to believers, except for a low collective responsibility), “middlemen” (14.6%; middle in all 5C constructs), and “contradictors” (3.7%; high in all 5C constructs). Compared to believers, middlemen were younger, more likely to be female, childless, less educated, held lower professional titles, had fewer years of nursing service, sometimes or never complied with recommended vaccinations, had satisfactory or poor self-assessed health status, had no work experience during the COVID-19 epidemic, and possessed greater levels of knowledge. Free riders were more likely to work in community health centers and have a lower degree than believers. Contradictors were more likely to work in community health centers, had junior college degrees or lower, and had no work experience during the COVID-19 epidemic than believers. From the highest to the lowest on vaccination intention and attention to vaccine news were believers, then free riders, contradictors, and finally middlemen.

**Conclusion:**

This study could aid in the development of personalized vaccination strategies based on nurses' vaccine hesitancy profiles and predictors. In addition to vaccine believers, we identified other three profiles based on their 5C psychological antecedents, emphasizing the significance of establishing tailored vaccination campaigns. Further research into the prevalence of profile structure in other groups of healthcare workers is required.

## Introduction

The coronavirus disease 2019 (COVID-19) pandemic, which is caused by severe acute respiratory syndrome coronavirus 2 (SARS-CoV-2), poses a significant threat to global public health. Since 2019, over 7.6% (576 million) of the global population has been infected with SARS-CoV-2, resulting in over 6 million deaths (1). SARS-CoV-2 infection imposes a substantial cost on human health, including musculoskeletal health complaints (2) and low back pain (3) during the acute phases, tachycardia (4), mental health disorders (5), and other sequelae during the post-acute phase. This has necessitated that health services face the dual task of managing with the increase in acute infections and providing care for COVID-19 survivors. Vaccination is a critical step in achieving COVID-19 herd immunity safely (6). The most recent research indicates that the COVID-19 vaccine is still effective in preventing moderate to severe illness and death brought on by modern variants of problems like Delta and Omicron (7, 8). However, the vaccine has not been well received and varies greatly around the world. For instance, in Central Asia, Eastern Europe, and Africa, vaccine hesitancy for the COVID-19 vaccine is more pronounced (9). Consequently, it is critical to advocate for initiatives to expand vaccination programs and increase vaccine uptake, particularly in nations and populations with low vaccine uptake and significant vaccine hesitancy (10).

Vaccine hesitancy, according to the Strategic Advisory Group of Experts (SAGE) Working Group, is defined as a delay in accepting or refusing vaccination despite the availability of vaccination services (11). More than 90% of the 194 member countries of WHO reported vaccine hesitancy during 2015–2017 (12). Vaccine hesitancy can result in lower vaccination rates, allowing for a recurrence of vaccine-preventable diseases, ultimately jeopardizing the effectiveness of immunization efforts (13). Due to the serious risks it poses to public health, the WHO listed vaccine hesitancy as one of the top 10 global health threats for 2019 (14).

Although the reasons for vaccine hesitancy differ by country and population, healthcare workers play a critical role in restoring public trust in vaccines (15) and are frequently viewed as the group with the most influence over people's vaccination (16). Nurses are not only responsible for vaccination but also spend a significant amount of time providing vaccine knowledge and health education to patients (17), and they play a critical role in promoting vaccination and reducing vaccine hesitancy in all populations (16–18). Nurses have the most direct contact with patients of any healthcare workers, and they are typically more directly confronted with the public's vaccine apprehension. However, recent studies have shown that nurses are even more hesitant about vaccines than other health professionals in Singapore (nurses: 7.4% vs. physicians: 0%) (19), Chicago (nurses: 27.0% vs. physicians or advanced practitioners: 1.7%) (20), Cape Town (nurses: 49.2% vs. physicians: 10.2%) (21), and Kuwait (nurses: 29.2% vs. physicians: 9.6%) (22). In fact, the issue of high vaccine hesitancy rates among nurses can no longer be ignored according to the data in Turkey (68.6%), Hong Kong (63%), and Israel (61%) (23–25). Vaccine hesitancy can have a negative impact on nurses' health and influence their vaccine recommendation behavior to patients, as well as enhance patients' fears and suspicions about vaccination (16).

As a complicated and dynamically shifting term, vaccine hesitancy challenges the traditional perspective of a simple dichotomization of an individual's immunization behavior into acceptance or refusal (26). Previous findings support the need for focused communication actions to address vaccine hesitancy among certain populations in various geographic cultures (27, 28). Recent studies have also classified people into subgroups depending on their vaccination beliefs, such as hesitant, confident, and trade-off clusters (29), or believers, skeptics, outsiders, contradictors, and middler profiles (30). In our study, we used the 5C model to understand the psychological antecedents of vaccination among nurses (31), which includes five dimensions of confidence (trust in vaccine efficacy, safety, and necessity, as well as in the system providing the vaccine), complacency (perception of low disease risk), constraints (perception of low vaccine availability, affordability, and accessibility), the calculation (participation in information search), and collective responsibility (willingness to vaccinate to protect others through herd immunity).

The local COVID-19 epidemic in Shanghai has been rapidly spreading since March 2022 (32), and nursing staff has become the backbone of epidemic prevention and control. Although substantial research has been carried out on vaccine hesitancy, no single study exists that adequately investigates vaccine hesitancy profiles among nurses in mainland China. Latent profile analysis (LPA) is a person-centered algorithm that will examine and identify unobserved heterogeneity in a population of nurses with vaccine hesitancy (33). In this quantitative study with an online cross-sectional survey among Shanghai nurses, we aimed to identify the following research questions: ① conduct a potential profile analysis of the psychological antecedents of nurses' vaccine hesitancy in Shanghai by LPA; ② investigate how different predictor variables predicate the profiles to which nurses belong; and ③ investigate how nurses in different profiles differ in their intentions to uptake the COVID-19 vaccine and attention to COVID-19 vaccine news.

## Study methods

An exploratory, cross-sectional latent profile analysis (LPA) on vaccine hesitancy was conducted among nurses in Shanghai, China. Ethics approval was granted by the Institutional Review Board of the School of Public Health and Nursing at Shanghai Jiao Tong University (Reference number: SJUPN-202018).

### Participants and data collection

Nurses from Shanghai's tertiary hospitals and community health centers (CHCs) participated in this study before the beginning of the COVID-19 booster vaccination program in China. Researchers contacted several hospitals and partnering community health centers affiliated with Shanghai Jiao Tong University School of Medicine, and nurses who volunteered to provide data for the study were recruited through advertisements. The pilot survey was first conducted in May 2021, before the formal conduct of the study. A purposive sample of 10 nurses from Shanghai was selected for the pre-survey of the study instrument. By recording the respondents' level of understanding of the content and format of the questionnaire and suggestions for modifications, we adjusted for specific situations to improve the accuracy and clarity of the questionnaire. From July to August 2021, nurses who were interested in participating in the study completed an online survey. No financial incentives are offered, and participation is entirely voluntary. We collected data *via* the Wenjuanxing website, and all participants were required to scan a QR code and provide informed consent on the survey platform before completing the questionnaire. Simplified Chinese is the language used in the questionnaire. A total of 2017 nurses completed the survey, and a final sample of 1928 was included for analysis, after deleting invalid responses. Inclusion criteria were that participants were (1) working nurses and (2) not nursing trainees or practical nurses.

### Questionnaire composition

#### Demographic characteristics

Participants were requested to give sociodemographic information in the first section of our study, including age (<30 years, ≥30 years), sex (male, female), marital status (unmarried, married), no. of children (0, ≥1), workplace (tertiary hospital, community health center), education level (junior college degree or lower, bachelor degree or higher), professional title (nurse or senior nurse, supervisor or professor nurse), years of nursing service (0–10, >10), previous compliance with recommended vaccination (sometimes or never, always), chronic diseases (yes, no), self-assessment of health status (very good or good, satisfactory or fair), and working experience during COVID-19 epidemic (yes, no).

#### Psychological antecedents of vaccine hesitancy

A questionnaire based on the 5C scale was used to assess the psychological antecedents of vaccine hesitancy. The 5C scale consists of 15 items, including five subscales consisting of three items each, with subscales addressing each of the five psychological antecedents: confidence, complacency, constraint, calculation, and collective responsibility. For these items, the allowable response values range from 1 to 7 (1 = strongly disagree; 7 = strongly agree). For each subscale, average scores were generated; the higher the mean value, the more consistent the associated region is in that construct. The higher mean value of the construct indicates stronger consistency of that construct. While the original 5C scale was designed to assess vaccinations in general, we added prompts before participants completed the section to make it vaccine-specific and to focus on the COVID-19 vaccine specifically.

Since the original scale was developed in English, the Chinese version of the 5C scale was developed through cross-cultural adaptation and psychometric testing after gaining allowed approval from the original authors. The 5C scale was translated from English to Chinese using Brislin's translation approach (34). A further validation process was implemented by exploratory and confirmatory factor analysis (EFA and CFA). According to the results of the parallel study, five factors should be kept in the vaccine hesitancy measurement. KMO measure (0.888) and Bartlett's test of sphericity (χ^2^ = 7729.676, *P* < 0.001) further confirmed the decomposability and sufficiency of the data sample, according to EFA results. Except for the backward scoring item that was part of the collective responsibility subscale of the original scale entered into the constraint subscale, all items conformed to the original factor structure using the Oblimin rotation, with factor loadings ranging from 0.577 to 0.912. As a result, the lone reverse item was put into the constraint subscale, and the original scoring was used to create the modified Chinese 5C scale, which gave a 5-factor structure that explained 77.908 % of the total variance. The redesigned scale's CFA (*X*^2^)/*df* ration indicates good agreement with 2.73, while TLI (0.929), CFI (0.946), and RMSEA (0.081) goodness-of-fit indices demonstrated good fit. Supplementary material shows the detailed process.

#### Knowledge level of COVID-19 vaccine and vaccination

A questionnaire was developed based on the COVID-19 vaccination knowledge on the technical guidelines and expert consensus. A focus group discussion was held to choose and revise the questionnaire's items after the first draft was finished. The discussion convened two chief physicians from the Department of Infection, one chief physician from the Department of Respiratory Medicine, and two professors from the School of Public Health. After that, a pilot study revisited the updated questionnaire. A random sample of 30 nurses was pre-surveyed before the survey's official launch to ensure the questionnaire's internal consistency. The Cronbach's coefficient was 0.732. In all, the final questionnaire had 30 closed-ended items (which included vaccine type, recommended immunization practices, recommendations for populations, adverse effects, and misunderstandings) that could be answered with a simple “yes” or “no.” The accurate response rate (a possible range of = 0.0–100.0%) was used to measure participants' knowledge of the COVID-19 vaccination. The correct response rates were divided into two categories: pass (≥60%) and fail (<60%).

#### Vaccine-related outcomes

Vaccine-related outcomes include two indicators of vaccination intention and attention to the news. The intention to take the COVID-19 vaccine was measured by a single item that asked participants on a Likert scale (0 = complete refusal; 5 = complete agreement) how likely they would be to have the COVID-19 vaccine when it is recommended for the current vaccination schedule. One question was utilized to evaluate the participants' attention to news reports about the COVID-19 vaccination. The item was assessed on a five-point Likert scale ranging from 1 (do not care at all) to 5 (care a great deal), with higher scores indicating greater interest in vaccine information.

### Statistical analysis

Person-centered analysis approach, in contrast to “variable-centered” statistical methods that treat individuals as homogeneous or essentially homogeneous, focuses on studying combinations or developmental patterns of behavioral variables to produce more individually meaningful statistical results. It has been used in health and psychological behavioral research, for example, in examining the profiles of emotional labor (35), vulnerability types (36), and symptoms pattern of fatigue (37). For the objective of determining the antecedents of vaccine hesitancy, person-centered analysis would be the most appropriate sort of statistical technique. The most basic and often used approaches in this study are latent class analysis (LCA) and latent profile analysis (LPA). Latent profile analysis is to categorize individuals based on their response patterns to epiphenomenal items, allowing for the investigation of diverse groups of population attributes. The potential profile analysis (38) was used to examine the number of unobserved categories (i.e., categorical potential profiles of vaccine hesitancy), characterize the properties of the classes, and calculate the probability that each individual belongs to a given class, given that the 5C scale entries were transformed into continuous variables (39).

In the latent profile analysis, the average scores of the five dimensions of vaccine hesitancy were used as the exogenous variables to develop the model. Starting with a model with one potential class, the number of potential classes was gradually increased, and the fitness of each model was evaluated one by one to determine the best potential class model. To compare models with different numbers of classes, the Lo-Mendell-Rubin likelihood ratio test (LMR) (40) and the bootstrap likelihood ratio test (BLRT) (38) were employed as significant tests. The model with k classes is superior to the model with k~1 classes if the LMR or BLRT is significant (*P* < 0.05) (41). Among the LPA model fit test measures are the Akaike Information Criterion (AIC), Bayesian Information Criterion (BIC), and sample size–adjusted Bayesian Information Criterion (sBIC). Usually, the lower the AIC, BIC, and sBIC values in the model, the better it fits in comparison with the previous model (42). The entropy value is frequently used to assess the classification quality of the model, and >0.80 indicates that the classification accuracy surpasses 90% (43). In addition to considering the model's fitness, the ideal model should be based on theory, integrated with previous studies, and the interpretability of data results (44).

Sociodemographic characteristics (age, sex, marital status, children, workplace, education level, professional title, years of nursing service, previous vaccination habits, chronic diseases, and working experience during the COVID-19 epidemic) and COVID-19 vaccination knowledge level were used as predictor variables, the COVID-19 vaccination intention and attention to COVID-19 vaccine news were used as outcome variables, and we utilized the R3STEP and DCON commands in Mplus to model the predictors and outcomes of the latent categorical variable (45, 46). Scores on the 5C scale did not meet the normal distribution criteria, so the median (M) and interquartile range (P25, P75) were utilized to describe them and assess them nonparametrically. Correlation analysis was carried out using Spearman's correlation coefficient *rho*. Multiple group differences were evaluated using the Kruskal–Wallis test and reported *p*-values were adjusted to account for multiple comparisons using the Bonferroni *post-hoc* test. SPSS (version 26.0) and Mplus (version 8.3) were used to analyze the data. There were no missing values discovered.

## Results

### Participants and correlations among variables

In this online survey, a total of 2,017 questionnaires were completed; 65 were eliminated for the following reasons: The questionnaire was unfinished (*n* = 10), or the response time was too short (*n* = 55). Unlike prior research, this study included a certain number of community nurses (*n* = 718), more representative of the nurse population, and some of the participants (*n* = 343) worked as frontline nurses during the COVID-19 epidemic. Because the 5C scales vary in their theoretical predictive aspects of vaccination intention, we checked questionnaires with repeated responses in 15 entries in extreme cases, including responses with repeated 1 (*n* = 5), 2 (*n* = 2), 6 (*n* = 3), or 7 (*n* = 11). We finally retained 1,928 cases for subsequent analysis. The characteristics of the study sample are shown in Table 1.

**Table 1:** Demographic characteristics of the participating nursing staff (*N* = 1,928).

**Characteristic**	**Number (%)**
**Age (years)**	
20–30	909 (47.1%)
>30	1,019 (52.9%)
**Sex**	
Male	74 (3.8%)
Female	1,854 (96.2)
**Marital status**	
Unmarried	681 (35.3%)
Married	1,247 (64.7%)
**No. of children**	
0	904 (46.9%)
≥1	1,024 (53.1%)
**Workplace**	
Tertiary hospital	1,210 (62.8%)
Community health center	718 (37.2%)
**Educational level**	
Junior college degree or lower	608 (31.5%)
Bachelor degree or higher	1,320 (68.5%)
**Professional title**	
Nurse or senior nurse	1,319 (68.4%)
Supervisor or professor nurse	609 (31.6%)
**Years of nursing experience**	
0–10	1,048 (54.4%)
>10	880 (45.6%)
**Previous compliance with recommended vaccination**	
Sometimes or never	750 (38.9%)
Always	1,178 (61.1%)
**Chronic disease**	
Yes	196 (10.2%)
No	1,732 (89.8)
**Self-assessment of health status**	
Very good or good	530 (27.5%)
Satisfactory or fair	1,398 (72.5%)
**Working experience during COVID-19 epidemic**	
No	1,585 (82.2%)
Yes	343 (17.8%)
**Vaccine-related knowledge level**	
Fail	771 (40.0%)
Pass	1,157 (60.0%)

Correlations of study variables, including 5C vaccine hesitancy indicators and outcome variables, are shown in Table 2. On the seven-point Likert scale, participants had high scores in confidence (Median = 6.33, IR = 1.67), calculation (Median = 6.00, IR = 2.00), and collective responsibility (Median = 6.50, IR = 2.00) and low scores in complacency (Median = 2.67, IR = 2.67) and constraint (Median = 1.25, IR = 1.50). As expected, all 5C indicators were correlated with each other and all were significantly associated with COVID-19 vaccine intention. However, a positive correlation was calculated with vaccination intention (*r* = 0.118, *p* < 0.01), contradicting the original authors' hypothesis (31) but matching a study in the Hong Kong nurse population (30). In addition, the same pattern was detected for the frequency of paying attention to COVID-19 vaccine news.

**Table 2:** Correlations of 5C indictors and outcome variables (*N* = 1,928).

	**5C vaccine hesitancy indictors**					**Outcomes**	
	**Confidence**	**Complacency**	**Constraints**	**Calculation**	**Collective responsibility**	**Intention to COVID-19 vaccination**	**Attention to COVID-19 vaccine news**
Median ± IR	6.33 ± 1.67	2.67 ± 2.67	1.25 ± 1.50	6.00 ± 2.00	6.50 ± 2.00		
Mean ± SD						4.55 ± 0.97	4.36 ± 0.87
Range	1–7	1–7	1–7	1–7	1–7	0–5	1–5
Confidence	1	−0.247^**^	−0.454^**^	0.397^**^	0.503^**^	0.307^**^	0.318^**^
Complacency		1	0.586^**^	−0.242^**^	−0.235^**^	−0.159^**^	−0.157^**^
Constraints			1	−0.315^**^	−0.377^**^	−0.268^**^	−0.274^**^
Calculation				1	0.502^**^	0.118^**^	0.267^**^
Collective responsibility					1	0.203^**^	0.280^**^
Intention to COVID-19 vaccination						1	0.176^**^
Attention to COVID-19 vaccine news							1

SD, standard derivation; IR, interquartile range.

** p < 0.01.

### Model selection

**Figure 1:**
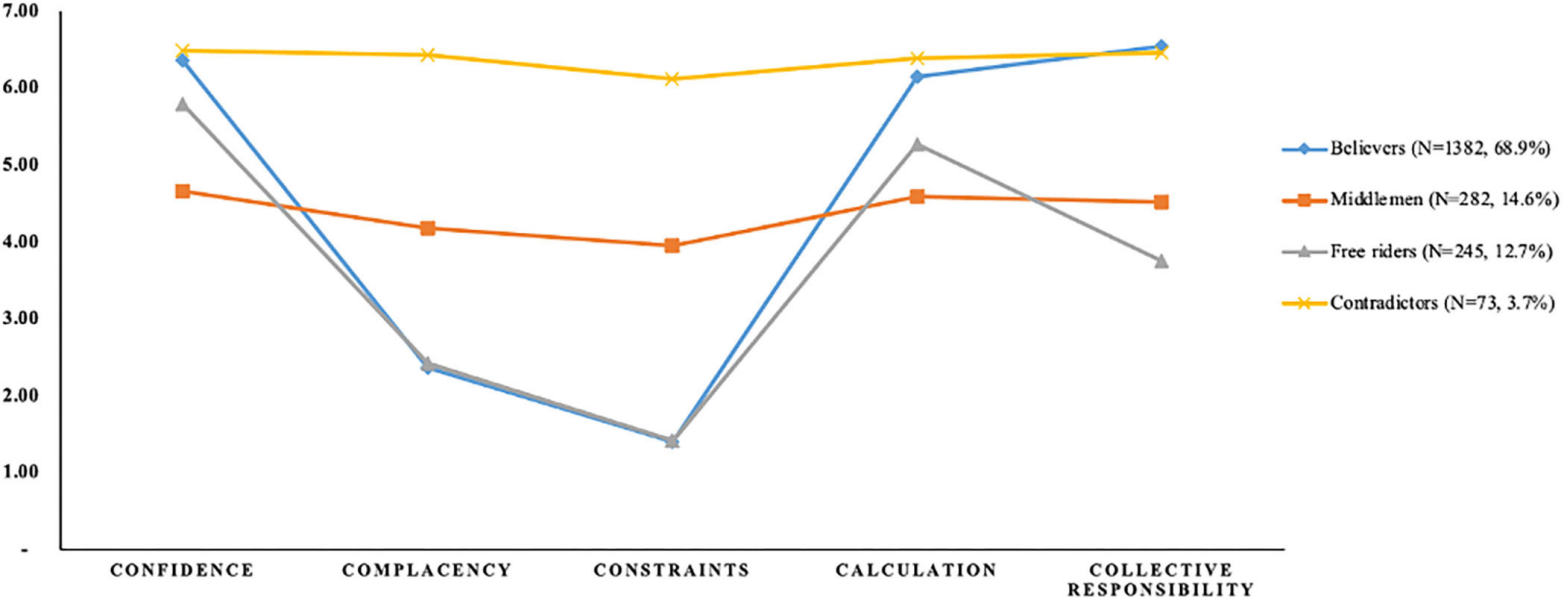
Four latent profiles with various levels of 5C indicators.

Starting with the initial model, one to six profile classes were modeled progressively when examining the data, and Table 3 shows the fitted statistics for the various latent profile structures. When five classes were retained, the information evaluation indexes AIC, BIC, and BIC decreased as the number of classes rose, the entropy values were optimal and LMR values reached significant levels. However, when five or more classes were kept, a smaller profile formed, accounting for <1% of the overall sample. Considering profiles of this site may be false (47), we did not investigate solutions with seven or more profiles further. According to the actual situation, more classes may disperse the information and result in false findings; therefore, a classification model with four profile classes is most fair (see Figure 1).

**Table 3:** Fit statistics for profile structures.

**Model**	**AIC**	**BIC**	**sBIC**	**LMR(*p*)**	**BLRT(*p*)**	**Entropy**	**Proportion of sample size in profile**
1 profile	32,443	32,499	32,467	–	–	–	1.000
2 profiles	30,175	30,264	30,213	0.0000	0.0000	0.943	0.825/0.175
3 profiles	29,232	29,354	29,284	0.0000	0.0000	0.958	0.159/0.802/0.038
4 profiles	28,635	28,790	28,701	0.0001	0.0000	0.927	0.127/0.146/0.689/0.037
5 profiles	28,318	28,318	28,210	0.0000	0.0000	0.941	0.127/0.155/0.675/0.006/0.037
6 profiles	27,898	27,898	27,771	0.0000	0.0000	0.915	0.106/0.119/0.581/0.005/0.155/0.034

AIC, Akaike information criteria; BIC, Bayesian information criteria; sBIC, sample size–adjusted BIC; LMR, Lo-Mendell-Rubin likelihood ratio test; BLRT, bootstrapped likelihood ratio test.

### Research question 1: Profile characteristics

Chi-square tests (Supplementary Table S1) showed that there was a significant difference in the four profiles for age (χ^2^ = 11.836, *p* = 0.008), workplace (χ^2^ = 38.495, *p* < 0.001), educational level (χ^2^ = 16.914, *p* = 0.001), professional title (χ^2^ = 19.622, *p* < 0.001), previous compliance with recommended vaccination (χ^2^ = 11.649, *p* = 0.009), self-assessment of health status (χ^2^ = 22.671, *p* < 0.001), working experience during COVID-19 epidemic (χ^2^ = 16.307, *p* = 0.001), and vaccine-rated knowledge level (χ^2^ = 11.994, *p* = 0.007). However, there was no significant difference in the three subtypes for gender, marital status, no. of children, and chronic disease. When compared with those in the other profiles, nurses in the “believers” subtype tended to be those who were >30 years, those who worked in tertiary hospitals, those who had more than undergraduate degrees, supervisor or professor nurse professional titles, better previous compliance with recommended vaccination, and better self-assessment of health status, and those who worked during COVID-19 epidemic, and better vaccine-related knowledge level.

Table 4 shows the distribution of 5C indicators between four profiles. Participants with high confidence (Median = 6.67), collective responsibility (Median = 7.00), and calculation (Median = 6.33) low complacency (Median = 2.33) and constraints (Median = 1.00) were labeled as believers (*N* = 1,382, 68.9%), which was the profile of the largest portion. Believers are most likely to be vaccinated, and they will actively seek out vaccination issues, believing that vaccines are efficacious and provide optimum protection to the public. Beyond that, they have few restrictions on vaccination.

**Table 4:** Profile characteristics of participants' responses on 5C indicators (*N* = 1,928).

**5C indictors**	**Believers** **(*N* = 1,328),** **Median ±IR**	**Middlemen** **(*N* = 282),** **Median ±IR**	**Free riders** **(*N* = 245),** **Median ±IR**	**Contradictors** **(*N* = 73), Median ±IR**	***P*** ^**a**^**-value**			
					**Overall**	**Believers vs. Middlemen**	**Believers vs. Free riders**	**Believers vs. Contradictors**
Confidence	6.67 ± 1.00	5.00 ± 1.00	6.00 ± 2.00	6.67 ± 1.00	<0.001	<0.001	<0.001	1.000
Complacency	2.33 ± 2.00	4.00 ± 1.33	2.33 ± 2.17	7.00 ± 1.00	<0.001	<0.001	1.000	<0.001
Constraints	1.00 ± 0.75	4.00 ± 1.25	1.00 ± 0.75	6.00 ± 1.13	<0.001	<0.001	1.000	<0.001
Calculation	6.33 ± 1.33	4.67 ± 1.00	5.33 ± 2.17	6.33 ± 1.00	<0.001	<0.001	<0.001	0.945
Collective responsibility	7.00 ± 1.00	4.50 ± 1.00	4.00 ± 1.00	7.00 ± 1.00	<0.001	<0.001	<0.001	1.000

IR, interquartile range.

There was also a profile marked as middlemen (*N* = 282, 14.6%), with all indicators around the sample median (Median _confidence_ = 5.00, Median _complacency_ = 4.00, Median _constraints_ = 4.00, Median _calculation_ = 4.67, Median _collectiveresponsibility_ = 4.50). They have mixed feelings about the efficacy of vaccines and the hazards of preventable diseases. They are apprehensive about the risks linked with vaccination, even though they can seek information and certify the herd immunity impact of vaccines to some level.

We marked high confidence (Median = 6.00) and calculation (Median = 5.33) and low other indicators (Median _complacency_ = 2.33, Median _constraints_=1.00, Median _collectiveresponsibility_ = 4.00) as free riders (N=245, 12.7%). They could search for information in response to vaccination questions, and they believed that vaccines are effective and had low limitations on vaccination. However, if others supply adequate protection, they could enjoy indirect protection as beneficiaries without contributing to herd immunization.

The profiles with the smallest part are contradictors (*N* = 73, 3.7%). They are high in all 5C indicators (Median _confidence_ = 6.67, Median _complacency_ = 7.00, Median _constraints_ = 6.00, Median _calculation_ = 6.33, Median _collectiveresponsibility_ = 7.00). Contradictors will conduct considerable research on vaccine-related topics, and while they recognize that vaccinations are helpful, they do not believe they need vaccines to stay healthy, or they may have too many barriers to vaccination. Furthermore, they consider that immunizations do protect the population.

### Research question 2: Predictors

Multinomial logistic regression was used to determine the predictors of nursing staff vaccine hesitancy profiles. Using the “believers” profile as the base outcome (reference), we obtained the following results (Table 5). We found that middlemen were younger, more likely to be female, had no children, had junior college degrees or lower, had lower professional titles, had fewer years of nursing service, sometimes or never complied with recommended vaccinations, had satisfactory or poor self-assessed health status, had no work experience during the COVID-19 epidemic, and had higher levels of knowledge than believers. Compared with believers, free riders were more likely to work in community health centers and had junior college degrees or lower. Contradictors were more likely to work in community health centers, had junior college degrees or lower, and had no work experience during the COVID-19 epidemic.

**Table 5:** Predicting pattern membership from individual characteristics.

**Variables**	**Middlemen**			**Free riders**			**Contradictors**		
	**β**	**OR (95% CI)**	***p-*** **value**	**β**	**OR (95% CI)**	***p-*** **value**	**β**	**OR (95% CI)**	***p-*** **value**
**Age (ref:20–30 years)**									
>30 years	−0.436	0.65 (0.49, 0.85)	0.002^**^	−0.102	0.90 (0.66, 1.24)	0.525	−0.400	0.67 (0.41, 1.09)	0.105
**Sex (ref: female)**									
Male	−0.724	0.49 (0.27, 0.87)	0.016^*^	−0.344	0.71 (0.33, 1.53)	0.379	0.965	2.63 (0.29, 23.50)	0.388
**Marital status (ref: unmarried)**									
Married	−0.086	0.92 (0.69, 1.22)	0.548	−0.044	0.96 (0.69, 1.33)	0.793	0.098	1.10 (0.56, 1.84)	0.709
**No. of children (ref: 0)**									
≥1	−0.324	0.72 (0.55, 0.95)	0.019^*^	−0.225	0.80 (0.58, 1.09)	0.159	0.055	1.06 (0.65, 1.71)	0.824
**Workplace (ref: community health center)**									
Tertiary hospital	−0.183	0.83 (0.63, 1.10)	0.200	−0.478	0.62 (0.45, 0.85)	0.003^**^	−1.411	0.24 (0.15, 0.41)	<0.001^***^
**Educational level (ref: junior college degree or lower)**									
Bachelor degree or higher	−0.361	0.70 (0.53, 0.93)	0.013^*^	−0.340	0.71 (0.51, 0.99)	0.042^*^	−0.781	0.46 (0.28, 0.74)	0.002^**^
**Professional title (ref: nurse or senior nurse)**									
Supervisor or professor nurse	−0.705	0.49 (0.36, 0.69)	<0.001^***^	−0.080	0.92 (0.66, 1.29)	0.639	−0.384	0.68 (0.40, 1.18)	0.168
**Years of nursing experience (ref: 0–10 years)**									
≥10 years	−0.504	0.60 (0.46, 0.80)	<0.001^***^	−0.180	0.84 (0.61, 1.14)	0.262	−0.349	0.71 (0.43, 1.15)	0.164
**Pervious compliance with recommended vaccination (ref: sometimes or never)**									
Always	−0.471	0.62 (0.48, 0.82)	0.001^**^	−0.078	0.93 (0.67, 1.28)	0.635	−0.115	0.89 (0.55, 1.46)	0.648
**Chronic disease (ref: no)**									
Yes	0.126	1.13 (0.73, 1.75)	0.572	0.041	1.04 (0.62, 1.75)	0.878	0.255	1.29 (0.62, 2.69)	0.496
**Self–assessment of health status (ref: very good or good)**									
Satisfactory or fair	0.840	2.32 (1.61, 3.33)	<0.001	0.233	1.26 (0.88, 1.80)	0.200	−0.009	0.99 (0.59, 1.67)	0.975
**Working experience during COVID-19 epidemic (ref: no)**									
Yes	−0.556	0.57 (0.39, 0.85)	0.006^**^	−0.380	0.68 (0.44, 1.06)	0.089	−1.252	0.29 (0.11, 0.74)	0.010^*^
**Vaccine-related knowledge level (ref: pass)**									
Fail	−0.460	0.63 (0.48, 0.83)	0.001^**^	0.059	1.06 (0.77, 1.47)	0.720	0.016	1.02 (0.62, 1.67)	0.951

Values in the table are estimates from the R3STEP logistic regression analyses using Mplus. “Believers” is the reference category.

* p < 0.05,

** p < 0.01,

*** p < 0.001.

### Research question 3: Outcomes

The COVID-19 vaccine-related outcomes showed the following results (see Table 6). The highest intentions for taking the COVID-19 vaccine when recommended were reported by believers (M = 4.697) and contradictors (M = 4.632), who did not significantly differ from one another. In comparison with all other profiles, middlemen had a significantly lower intention to uptake the COVID-19 vaccine (M = 3.964). A similar pattern can be observed for the frequency of paying attention to COVID-19 vaccine news. Believers (M = 4.505) and contradictors (M = 4.497) reported a significantly higher frequency of paying attention to vaccine-related news across all profiles. Middlemen were having a significantly lower frequency of following vaccine-related news than all other classes (M = 3.752).

**Table 6:** Results of predicting outcomes of latent profile membership.

**Outcomes**	**Believers**		**Middlemen**		**Free riders**		**Contradictors**		**Overall χ^2^**	***p-*** **value**
	**Mean**	***SE***	**Mean**	***SE***	**Mean**	***SE***	**Mean**	***SE***		
Intention to COVID-19 vaccination	4.697 _bc_	0.020	3.964 _acd_	0.087	4.423 _ab_	0.069	4.632 _b_	0.097	77.841	*P* < 0.001
Attention to COVID-19 vaccine news	4.505 _bc_	0.020	3.752 _acd_	0.064	4.244 _ab_	0.056	4.479 _bc_	0.088	137.097	*P* < 0.001

Analyses were conducted using DCON command in Mplus. The subscript letters represent that the mean value of this profile was significantly different from the mean value of the profile labeled by the subscript letter. For example, the value 3.964 _acd_ indicates that the intention of taking the COVID-19 vaccine in Profile (b) was significantly different from Profile (a), Profile (c), and Profile (d).

## Discussion

Before the implementation of the booster vaccination program in China, this study focused on nursing staff to understand the heterogeneity of vaccine hesitators and to provide specific evidence for targeted interventions to address vaccine hesitancy. We found a profile that was high in both confidence and collective responsibility (believers), as expected, and another profile that was high in confidence but low in collective responsibility (free riders). There were two quantitatively distinct profiles, with individuals having all 5C constructs around the median (middlemen) and all at high levels (contradictors). The study also observed differences between profiles in terms of predictors, and the profiles revealed disparities in their intention to COVID-19 vaccination and attention to COVID-19 vaccine news.

In this study, nurses had higher median score in confidence (Median = 6.33), calculation (Median = 6.00), and collective responsibility (Median = 6.50) and lower median score in complacency (Median = 2.67) and constraints (M = 1.25). The overall distribution of the five dimensions is similar to prior research on nurses in Hong Kong (30). However, our findings contradict Betsch's (31) assumptions about the structure of the calculation. They expected that individuals with superior computational skills would evaluate the risk of infection and vaccinations to make the correct choice. Therefore, those with a high level of computing ability should be risk-averse, and those with a more careful decision-making process may have a lesser intention to vaccinate. However, there is evidence that those who seek further vaccine information are more likely to be vaccinated (48). People with good computing skills should be wary about taking risks, but the link between calculation and vaccination is unclear and still needs to be further explored in different cultural contexts.

### Contribution to the tailored interventions for the four profiles

Our study found that there are four types of nurses based on the 5C structure of vaccine hesitancy. Among them, the largest proportion was believers (68.9%), a group with the highest intention to vaccinate and the highest frequency of attention to vaccine-related information, which is very helpful for the smooth progress of vaccination. Therefore, it is necessary to find the differences between the other three profiles and believers and adopt targeted interventions.

Participants with all indicators around the sample median made up 14.6% of the population, who were categorized as middlemen. They had the lowest intention of taking the COVID-19 vaccine and frequency of paying attention to vaccine news than the other three profiles. The rapid spread of the COVID-19 pandemic forced people to rapidly acquire and implement health knowledge and change their behavior (49), and the calculations were highly correlated with perceptions of disease risk and vaccination risk (31). Compared to believers, middlemen have less confidence in the efficacy and safety of the COVID-19 vaccine and are less motivated to search for information about the vaccine with a sense of collective responsibility. While the emergence of multiple social media platforms has made it simpler to acquire more information regarding the COVID-19 vaccine and vaccination, new outbreak patterns and shifting health information have hindered the proper handling and utilization of health information during a COVID-19 pandemic (50). Although younger nurses may be more proficient at using social media to get information, their lack of education and work experience makes it difficult for them to spot vaccine rumors, which add to their reduced confidence in the COVID-19 vaccine. In addition, their lack of children, lack of vaccination history, perception of their health, and lack of direct work experience with the epidemic made them less concerned about the value of the vaccine for pandemic containment. Therefore, strengthening middlemen's trust in the COVID-19 vaccine and their capacity to locate important information is crucial for nurses to perform their job as health educators and prevent the spread of the pandemic both within the hospital and in the community.

Participants with high confidence but low collective responsibility accounted for 12.7% of the population, which were named free riders. It is clear from the results that free riders had a higher intention of taking the COVID-19 vaccine and frequency of paying attention to vaccine news than middlemen but were lower than the other two profiles. Collective responsibility appears to be a more fundamental factor in free riders' decisions to get the COVID-19 vaccine than in believers. People who believe in collective responsibility advocate for individual subordination to society and feel that the collective's interests trump the individuals, which implies they will participate in more pro-social conduct (51). Our study presents a very interesting result that nurses with low education and community nurses are more inclined to be free riders. This phenomenon can be explained by the fact that lower information-seeking ability is also a characteristic of this subgroup and that information-seeking ability is positively associated with collective responsibility. Much of the information in China about the COVID-19 vaccine emphasizes societal and governmental efforts to develop the vaccine, its safety and efficacy, and the significance of coordinated efforts to stop the pandemic (52). People acquire a strong belief in their own and society's responsibility for containing the spread of COVID-19 as they seek out more information about the COVID-19 vaccine from a variety of media sources (53). However, it is of concern to us that collectivists lack confidence in their decisions compared to individualists (54). Nurses with higher levels of collectivism may be more likely to regret their previous vaccination decisions than nurses with lower levels of collectivism. Therefore, providing more transparent information to enhance the credibility of the vaccine is as important as highlighting the specific societal benefits of vaccination for nurses who bear the risk of curbing COVID-19 infections (55).

The survey results demonstrate that, despite making up the smallest fraction of these four groups, the contradictors (3.7%) are not the least likely to be vaccinated and the least likely to follow vaccine news. This group possesses the same high levels of confidence, calculation, and collective responsibility as believers, but in contrast to believers, they also demonstrate a very high level of complacency and constraints. As a result, their perspectives on the advantages and hazards of vaccination are equivocal. This could indicate a lack of concern about the COVID-19 vaccine's function in curbing the spread of the epidemic, an undue complacency about their health status, or an unwillingness to confront the limits imposed on them by vaccination barriers. As a result, making health information more available and explaining the risk of developing the disease are extremely critical in persuading these healthcare providers to be vaccinated. Furthermore, workload and shift work are barriers to vaccination and particularly affect nurses' vaccination rates (56), and it is critical to equip them with flexible immunization schedules and locations.

### Implications of this study for the current situation and the future

For nurses themselves, vaccination is very important for their protection in high-risk settings. Even though the vaccination rate among Chinese nurses is high, their reluctance to uptake the COVID-19 vaccine is commonly disregarded, which may impede the advancement of continuous immunization programs. Nurses are not vaccinologists and do not know everything about vaccine development, clinical trials, etc. (57). They may not have enough information about vaccine efficacy and safety, but they are still very motivated to vaccinate for their protection and the protection of others, especially patients (58). Our study aimed to determine the psychological status of Chinese nurses regarding COVID-19 vaccination. In addition, to gain a deeper understanding, we abandoned previous studies that only explored the behavior of nursing staff to vaccinate or not to vaccinate, or the psychological state of hesitation or not to hesitate, and instead used a person-centered approach to understand the heterogeneity of nursing staff's vaccine hesitancy.

For patients and the public, our study is also relevant. Nursing staff are at the front line of safeguarding public health and are a reliable source of vaccine-related information (59), and many studies have demonstrated that pediatric nurses, obstetric and gynecological nurses, and community nurses play an important role in promoting vaccination and reducing vaccine hesitancy in different populations (17, 18, 60). Although not all nurses are directly responsible for vaccines, nurses spend far more time with patients than other medical personnel (17). Patients and the public view them as thought leaders; thus, their participation in vaccine-related health education should not be disregarded (16). They help patients understand the history and efficacy of vaccination by providing them with vaccine-related information and health education to promote public trust in vaccinations and decrease the frequency of vaccine hesitancy or refusal (60). In this study, believers had the highest readiness to vaccinate and the highest level of vaccine concern compared to the other three categories. These nurses would contribute tremendously to the seamless implementation of vaccination and immunization planning. Our findings therefore provide a factual foundation for an acceptable intervention to assist the other three subgroups of nurses who are hesitant about vaccines.

In addition, this study has other public health implications in promoting vaccination efforts. First, we found some association between the 5C model and vaccination intention among nurses in mainland China. In future, tailored immunization promotion interventions can also be developed based on testing the psychological antecedents of vaccination in other groups of healthcare workers or even the public. Second, this study was conducted before the third dose (booster) of the COVID-19 vaccine in Chinese adults. Since the COVID-19 pandemic is likely to be widespread over a long period, a person-centered approach to vaccine hesitancy at different time points in the pandemic could help control the social and economic impact of the pandemic (61). Third, this study found that it is important to further improve the science of evidence-based risk-benefit assessment of vaccines. Public communication pathways and models regarding vaccine efficacy and safety should also be actively explored in the promotion of vaccination campaigns for other vaccine types, not just for the COVID-19 vaccine, and public transparency of information should be enhanced to boost public confidence in vaccines.

## Limitation

Despite the practical implications of the results of this study, there are some limitations to its generalizability. First, we used convenience sampling, which inhibits generalizability. Future studies should investigate samples from a variety of other settings to further analyze the characteristics of nurses' hesitancy to work with vaccines in the Shanghai region vs. other provinces and cities. Second, we implemented a cross-sectional design, and vaccine hesitancy is susceptible to pandemic severity. Therefore, longitudinal studies are needed to explore the long-term changes in vaccine hesitancy and the factors influencing it. Third, since participants may answer these items in a manner consistent with social expectations, the results may be biased. Fourth, our choice of the 5C model as a theoretical framework to understand participants' vaccine hesitancy issues for COVID-19 was not completed adequately, so some others such as vaccine literacy and altruistic beliefs (62, 63) can be added in future studies.

## Conclusion

Overall, Shanghai nurses demonstrated a high level of confidence, calculation, collective responsibility, low complacency, and constraints with COVID-19 vaccination. By profiling the psychological antecedents of COVID-19 vaccination among nurses in Shanghai, this study identified four distinct profiles of vaccine hesitancy related to COVID-19 (named “believers,” “free riders,” “middlemen,” and “contradictors”). We further explored the differences in sociodemographic, vaccine knowledge, vaccination intention, and attention to vaccine news among individuals between each profile. The characteristics of the latent profiles can help provide more targeted guidance for nursing managers to develop interventions that complement vaccine knowledge gained through continuing education, provide some peer or supervisory support, and thus aid nurses in reducing vaccine hesitancy and facilitating smooth vaccination and immunization planning.

## Data availability statement

The original contributions presented in the study are included in the article/Supplementary material, further inquiries can be directed to the corresponding author.

## Ethics statement

The studies involving human participants were reviewed and approved by the Institutional Review Board of the School of Public Health and Nursing at Shanghai Jiao Tong University (Reference number: SJUPN-202018). Participants provided informed consent to participate in the study by using an electronic informed consent form.

## Author contributions

EZ: conceptualization, data curation, formal analysis, and writing—original draft. ZD: conceptualization, data curation, investigation, and writing—reviewing and editing. CW: conceptualization and writing—reviewing and editing. JH: writing—reviewing and editing. SW: conceptualization, data curation, and investigation. LZ: writing—reviewing and editing. QF: writing—reviewing and editing, project administration, and resources. All authors contributed to the article and approved the submitted version.

## Funding

This study was supported by grants from the three-year action plan for the construction of Shanghai's public health system (2020–2022), academic leaders cultivating project (Grant No. GWV-10.2-XD33), an Innovative research team of high-level local universities in Shanghai (Grant No. SHSMU-ZDCX20212801), and Shanghai Jiao Tong University School of Nursing Student Innovation Training Program (Grant No. HLDC21-05).

## Conflict of interest

The authors declare that the research was conducted in the absence of any commercial or financial relationships that could be construed as a potential conflict of interest.

## Publisher's note

All claims expressed in this article are solely those of the authors and do not necessarily represent those of their affiliated organizations, or those of the publisher, the editors and the reviewers. Any product that may be evaluated in this article, or claim that may be made by its manufacturer, is not guaranteed or endorsed by the publisher.
