# Supplementary material for: Targeting COVID-19 vaccine hesitancy among nurses in Shanghai: A latent profile analysis
Source: Front Public Health. 2022 Sep 14;10:953850. doi: 10.3389/fpubh.2022.953850 (PMC9515966; doi:10.3389/fpubh.2022.953850)
Supplement: Supplementary file 1 [file Data_Sheet_1.zip › Supplementary Material/Supplementary material 3.docx]

COVID-19 vaccination knowledge questionnaire

| 序号 | 条目内容 | 回答 |
| --- | --- | --- |
| 1 | 灭活疫苗是所有类型疫苗中最安全的 | “是”或“否” |
| 2 | 未按规定时间完成第2剂或3剂疫苗接种者，应重新开始免疫程序 | “是”或“否” |
| 3 | 新型冠状病毒与人类可能会长期共存，未来或需定期接种疫苗 | “是”或“否” |
| 4 | 当因动物致伤、外伤等原因需接种狂犬病疫苗、破伤风疫苗时，要与新冠疫苗的接种间隔大于14天 | “是”或“否” |
| 5 | 如果在接种新冠疫苗后怀孕或在未知怀孕的情况下接种新冠疫苗，应及时终止妊娠 | “是”或“否” |
| 6 | 接种新冠疫苗能有效预防新冠肺炎感染，接种后不会再感染 | “是”或“否” |
| 7 | 疫情现在控制的较好，可以等疫情严重时再接种 | “是”或“否” |
| 8 | 接种新冠疫苗后，则没有必要做核酸检测 | “是”或“否” |
| 9 | 完成新冠疫苗接种后，个人防护可以放松 | “是”或“否” |
| 10 | 一旦新冠病毒发生变异，疫苗则无保护作用 | “是”或“否” |
|  | 目前我国已经开展的新冠疫苗类型 |  |
| 11 | 灭活疫苗 | “是”或“否” |
| 12 | 重组蛋白疫苗 | “是”或“否” |
| 13 | 减毒流感病毒载体疫苗 | “是”或“否” |
| 14 | 腺病毒载体疫苗 | “是”或“否” |
| 15 | 核酸疫苗 | “是”或“否” |
|  | 目前，暂不推荐接种新冠疫苗的人群 |  |
| 16 | 60岁以上人群 | “是”或“否” |
| 17 | 18岁以下人群 | “是”或“否” |
| 18 | 孕期或哺乳期女性 | “是”或“否” |
| 19 | 既往新冠感染者 | “是”或“否” |
| 20 | 青霉素、头孢等抗生素过敏者 | “是”或“否” |
|  | 目前，暂不推荐接种新冠疫苗的情况 |  |
| 21 | 正在发热者 | “是”或“否” |
| 22 | 患有急性疾病者 | “是”或“否” |
| 23 | 慢性疾病的急性发作者 | “是”或“否” |
| 24 | 未控制的严重慢性病患者 | “是”或“否” |
| 25 | 癌症病情稳定、目前无需化疗或治疗者 | “是”或“否” |
|  | 属于疫苗接种的不良反应中异常反应的情况 |  |
| 26 | 发热、头痛、四肢关节痛 | “是”或“否” |
| 27 | 食欲不振、恶心、呕吐、腹泻 | “是”或“否” |
| 28 | 接种部位红晕或硬节 | “是”或“否” |
| 29 | 过敏性休克 | “是”或“否” |
| 30 | 器官或功能损害相关反应 | “是”或“否” |
