# Supplementary material for: Targeting COVID-19 vaccine hesitancy among nurses in Shanghai: A latent profile analysis
Source: Front Public Health. 2022 Sep 14;10:953850. doi: 10.3389/fpubh.2022.953850 (PMC9515966; doi:10.3389/fpubh.2022.953850)
Supplement: Supplementary file 1 [file Data_Sheet_1.zip › Supplementary Material/Supplementary material 2.docx]

**Table 1 Factor loadings of the Chinese version of 5C scale**

| **Factor** | | | | | |
| --- | --- | --- | --- | --- | --- |
| **Items** | **Confidence** | **Complacency** | **Constraints** | **Calculation** | **Collective responsibility** |
| Q1 | 0.924 | — | — | — | — |
| Q2 | 0.910 | — | — | — | — |
| Q3 | 0.848 | — | — | — | — |
| Q4 | — | 0.742 | — | — | — |
| Q5 | — | 0.897 | — | — | — |
| Q6 | — | 0.680 | — | — | — |
| Q7 | — | — | 0.738 | — | — |
| Q8 | — | — | 0.865 | — | — |
| Q9 | — | — | 0.845 | — | — |
| Q10 | — | — | — | 0.827 | — |
| Q11 | — | — | — | 0.895 | — |
| Q12 | — | — | — | 0.755 | — |
| Q13 | — | — | 0.660 | — | — |
| Q14 | — | — | — | — | 0.850 |
| Q15 | — | — | — | — | 0.795 |

**Fig 1 Confirmatory factor analysis of the 15 questions related to the 5 domains of 5C scale of vaccine antecedent.**


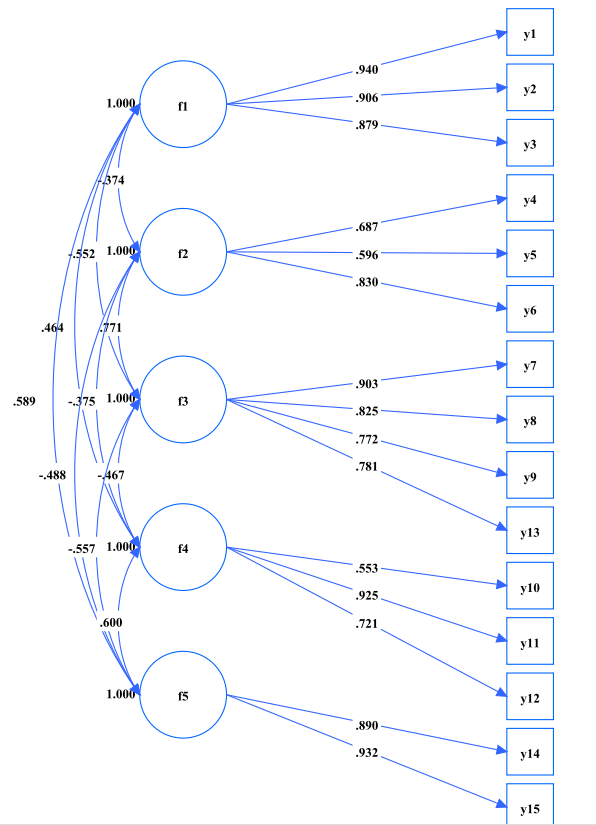


**Table 2 Reliability of the Chinese version of the 5C scale before adjustment.**

| **Dimension** | **Items** | **Cronbach’s alpha** |
| --- | --- | --- |
| **Confidence** | 3 | 0.929 |
| I am completely confident that vaccines are safe. |  |  |
| Vaccinations are effective. |  |  |
| Regarding vaccines, I am confident that public authorities decide in the best interest of the community. |  |  |
| **Complacency** | 3 | 0.773 |
| Vaccination is unnecessary because vaccine-preventable diseases are not common anymore. |  |  |
| My immune system is so strong, it also protects me against diseases. |  |  |
| Vaccine-preventable diseases are not so severe that I should get vaccinated. |  |  |
| **Constraints** | 3 | 0.853 |
| Everyday stress prevents me from getting vaccinated. |  |  |
| For me, it is inconvenient to receive vaccinations. |  |  |
| Visiting the doctor’s makes me feel uncomfortable; this keeps me from getting vaccinated. |  |  |
| **Calculation** | 3 | 0.787 |
| When I think about getting vaccinated, I weigh benefits and risks to make the best decision possible. |  |  |
| For each and every vaccination, I closely consider whether it is useful for me. |  |  |
| It is important for me to fully understand the topic of vaccination, before I get vaccinated. |  |  |
| **Collective responsibility** | 3 | 0.683 |
| When everyone is vaccinated, I don’t have to get vaccinated, too. (R) |  |  |
| I get vaccinated because I can also protect people with a weaker immune system. |  |  |
| Vaccination is a collective action to prevent the spread of diseases. |  |  |

**Table 3 Reliability of the Chinese version of the adjusted 5C scale.**

| **Dimension** | **Items** | **Cronbach’s alpha** |
| --- | --- | --- |
| **Confidence** | 3 | 0.929 |
| I am completely confident that vaccines are safe. |  |  |
| Vaccinations are effective. |  |  |
| Regarding vaccines, I am confident that public authorities decide in the best interest of the community. |  |  |
| **Complacency** | 3 | 0.773 |
| Vaccination is unnecessary because vaccine-preventable diseases are not common anymore. |  |  |
| My immune system is so strong, it also protects me against diseases. |  |  |
| Vaccine-preventable diseases are not so severe that I should get vaccinated. |  |  |
| **Constraints** | 4 | 0.866 |
| Everyday stress prevents me from getting vaccinated. |  |  |
| For me, it is inconvenient to receive vaccinations. |  |  |
| Visiting the doctor’s makes me feel uncomfortable; this keeps me from getting vaccinated. |  |  |
| When everyone is vaccinated, I don’t have to get vaccinated, too. (R) |  |  |
| **Calculation** | 3 | 0.787 |
| When I think about getting vaccinated, I weigh benefits and risks to make the best decision possible. |  |  |
| For each and every vaccination, I closely consider whether it is useful for me. |  |  |
| It is important for me to fully understand the topic of vaccination, before I get vaccinated. |  |  |
| **Collective responsibility** | 2 | 0.825 |
| I get vaccinated because I can also protect people with a weaker immune system. |  |  |
| Vaccination is a collective action to prevent the spread of diseases. |  |  |
