# Supplementary material for: Targeting COVID-19 vaccine hesitancy among nurses in Shanghai: A latent profile analysis
Source: Front Public Health. 2022 Sep 14;10:953850. doi: 10.3389/fpubh.2022.953850 (PMC9515966; doi:10.3389/fpubh.2022.953850)
Supplement: Supplementary file 1 [file Data_Sheet_1.zip › Supplementary Material/Supplementary material 1.docx]

**Actual questions and response options regarding the 5C psychological antecedents of vaccination (confidence, complacency, convenience, calculation, and collective responsibility) which were used in the survey, translated from Chinese to English.**

**Translation table-Initial version**

| **Translated version (Chinese)** | **English version** |
| --- | --- |
| **Confidence** | |
| 我有充分的信心认为疫苗是安全的 | **I am completely confident that vaccines are safe.** |
| 接种疫苗是有效的 | Vaccinations are effective. |
| 关于疫苗，我相信政府当局的决定会使社会公众的利益最大化 | Regarding vaccines, I am confident that public authorities decide in the best interest of the community. |
| **Complacency** | |
| 接种疫苗不是必要的，因为疫苗可预防性疾病不再普遍出现 | **Vaccination is unnecessary because vaccine-preventable diseases are not common anymore.** |
| 我的免疫系统非常强大，它能保护我免受疾病侵扰 | My immune system is so strong, it also protects me against diseases. |
| 疫苗可预防的疾病并没有严重到我应该接种疫苗的程度 | Vaccine-preventable diseases are not so severe that I should get vaccinated. |
| **Constraints** | |
| 日常压力会妨碍我接种疫苗 | **Everyday stress prevents me from getting vaccinated.** |
| 接种疫苗对于我来说是不方便的 | For me, it is inconvenient to receive vaccinations. |
| 看到医务人员的不舒适感让我无法接种疫苗 | Visiting the doctor’s makes me feel uncomfortable; this keeps me from getting vaccinated. |
| **Calculation** | |
| 当我考虑接种疫苗时，我会权衡益处和风险，尽可能做出最佳决定 | **When I think about getting vaccinated, I weigh benefits and risks to make the best decision possible.** |
| 对于每剂疫苗，我都会仔细考虑它对我是否有用 | For each and every vaccination, I closely consider whether it is useful for me. |
| 对我来说，在接种疫苗之前，充分了解疫苗接种信息对我来说很重要 | It is important for me to fully understand the topic of vaccination, before I get vaccinated. |
| **Collective responsibility** | |
| 当每个人都接种疫苗时，我也就不必接种疫苗 | **When everyone is vaccinated, I don’t have to get vaccinated, too. (R)** |
| 我会接种疫苗是因为可以保护免疫系统较弱的人 | I get vaccinated because I can also protect people with a weaker immune system. |
| 接种疫苗是一种预防疾病传播的集体行为 | Vaccination is a collective action to prevent the spread of diseases. |

Instruction: “Please evaluate how much you disagree or agree with the following statements.” (1=strongly disagree, 2=moderately disagree, 3=slightly disagree, 4=neutral, 5=slightly agree, 6=moderately agree, 7 strongly agree). Scoring: mean scores of each sub-scale. Item with (R) is reverse-coded. For the short scale use bold items.

提示：“请根据您对以下陈述的赞同或反对程度进行评估”（1=非常反对 2=较反对 3=略反对 4=中立 5=略赞同 6=较赞同 7=非常赞同）。计分方法：每个子量表的平均分。有（R）的条目代表反向计分。缩减版量表使用加粗条目。

**Translation table-final version**

| **Translated version (Chinese)** | **English version** |
| --- | --- |
| **Confidence** | |
| 我完全相信疫苗是安全的 | **I am completely confident that vaccines are safe.** |
| 疫苗接种是有效的 | Vaccinations are effective. |
| 政府相关部门关于疫苗的决定是从公众的最大利益出发的，对此我有信心 | Regarding vaccines, I am confident that public authorities decide in the best interest of the community. |
| **Complacency** | |
| 疫苗可预防的疾病不再常见，所以没有必要接种疫苗 | **Vaccination is unnecessary because vaccine-preventable diseases are not common anymore.** |
| 我的免疫系统非常强大，它能保护我免受疾病侵扰 | My immune system is so strong, it also protects me against diseases. |
| 疫苗可预防的疾病并未严重到我应该接种疫苗的程度 | Vaccine-preventable diseases are not so severe that I should get vaccinated. |
| **Constraints** | |
| 日常压力妨碍我接种疫苗 | **Everyday stress prevents me from getting vaccinated.** |
| 对于我来说，接种疫苗并不方便 | For me, it is inconvenient to receive vaccinations. |
| 去看医生让我感觉不舒服，这使我无法接种疫苗 | Visiting the doctor’s makes me feel uncomfortable; this keeps me from getting vaccinated. |
| **Calculation** | |
| 当考虑接种疫苗时，我会权衡益处和风险，尽可能做出最佳决定 | **When I think about getting vaccinated, I weigh benefits and risks to make the best decision possible.** |
| 对于每次接种疫苗，我都会仔细考虑它对我是否有用 | For each and every vaccination, I closely consider whether it is useful for me. |
| 在接种疫苗之前，充分理解疫苗接种信息对我来说很重要 | It is important for me to fully understand the topic of vaccination, before I get vaccinated. |
| **Collective responsibility** | |
| 当每个人都接种了疫苗，我也就不必接种了 | **When everyone is vaccinated, I don’t have to get vaccinated, too. (R)** |
| 我会接种疫苗，是因为这样也可以保护免疫系统较弱的人 | I get vaccinated because I can also protect people with a weaker immune system. |
| 疫苗接种是预防疾病传播的集体行为 | Vaccination is a collective action to prevent the spread of diseases. |

Instruction: “Please evaluate how much you disagree or agree with the following statements.” (1=strongly disagree, 2=moderately disagree, 3=slightly disagree, 4=neutral, 5=slightly agree, 6=moderately agree, 7 strongly agree). Scoring: mean scores of each sub-scale. Item with (R) is reverse-coded. For the short scale use bold items.

请根据您对以下陈述的赞同或反对程度进行评估”（1=非常反对 2=较反对 3=略反对 4=中立 5=略赞同 6=较赞同 7=非常赞同）。计分方法：每个子量表的平均分。有（R）的条目代表反向计分。缩减版量表使用加粗条目。
