# Supplementary material for: Targeting COVID-19 vaccine hesitancy among nurses in Shanghai: A latent profile analysis
Source: Front Public Health. 2022 Sep 14;10:953850. doi: 10.3389/fpubh.2022.953850 (PMC9515966; doi:10.3389/fpubh.2022.953850)
Supplement: Supplementary file 1 [file Data_Sheet_1.zip › Supplementary Material/Supplementary Table S1.docx]

**Supplementary Table S1**

| Characteristic | Believers | Middlemen | Free riders | Contradictors | Total | **χ ^2^** | *P* value |
| --- | --- | --- | --- | --- | --- | --- | --- |
| Age (years) |  |  |  |  |  | 11.836^a^ | 0.008** |
| 20-30 | 597 (45.0%) | 156 (55.3%) | 116 (47.3%) | 40 (54.8%) | 909 (47.1%) |  |  |
| >30 | 731 (55.0%) | 126 (44.7%) | 129 (52.7%) | 33 (45.2) | 1019 (52.9%) |  |  |
| Gender |  |  |  |  |  |  |  |
| Men | 44 (3.3%) | 18 (6.4%) | 11 (4.5%) | 1 (1.4%) | 74 (3.8%) | 7.426 ^a^ | 0.059 |
| Women | 1284 (96.7%) | 264 (93.6%) | 234 (95.5%) | 72 (98.6%) | 1854 (96.2) |  |  |
| Marital status |  |  |  |  |  | 0.583 ^a^ | 0.900 |
| Unmarried | 465 (35.0%) | 104 (36.9%) | 88 (35.9%) | 24 (32.9%) | 681 (35.3%) |  |  |
| Married | 863 (65.0%) | 178 (63.1%) | 157 (64.1) | 49 (67.1%) | 1247 (64.7%) |  |  |
| No. of children |  |  |  |  |  | 6.917 ^a^ | 0.075 |
| 0 | 600 (45.2%) | 149 (16.5%) | 123 (50.2%) | 32 (43.8%) | 904 (46.9%) |  |  |
| ≥1 | 728 (54.8%) | 133 (47.2%) | 122 (49.8%) | 41 (56.2%) | 1024 (53.1%) |  |  |
| Workplace |  |  |  |  |  | 38.495 ^a^ | <0.001*** |
| Tertiary hospital | 875 (65.9%) | 174 (61.7%) | 137 (55.9%) | 24 (32.9%) | 1210 (62.8%) |  |  |
| Community health center | 453 (34.1%) | 108 (38.3%) | 108 (44.1%) | 49 (67.1%) | 718 (37.2%) |  |  |
| Educational level |  |  |  |  |  | 16.914 ^a^ | 0.001** |
| <Undergraduate | 384 (28.9%) | 103 (36.5%) | 87 (35.5%) | 34 (46.6%) | 608 (31.5%) |  |  |
| ≥Undergraduate | 944 (71.1%) | 179 (63.5%) | 158 (64.5%) | 39 (53.4%) | 1320 (68.5%) |  |  |
| Professional title |  |  |  |  |  | 19.622 ^a^ | <0.001*** |
| Nurse or senior nurse | 876 (66.0%) | 223 (79.1%) | 166 (67.8%) | 54 (74.0%) | 1319 (68.4%) |  |  |
| Supervisor or professor nurse | 452 (34.0%) | 59 (20.9%) | 79 (32.2%) | 19 (26.0%) | 609 (31.6%) |  |  |
| Years of nursing experience |  |  |  |  |  | 14.201 ^a^ | 0.003** |
| 0-10 | 688 (51.8%) | 179 (63.5%) | 137 (55.9%) | 44 (60.3%) | 1048 (54.4%) |  |  |
| >10 | 640 (48.2%) | 103 (36.5%) | 108 (44.1%) | 29 (39.7%) | 880 (45.6%) |  |  |
| Previous compliance with recommended vaccination |  |  |  |  |  | 11.649 ^a^ | 0.009** |
| Sometimes or never | 491 (37.0%) | 135 (47.9%) | 95 (38.8%) | 29 (39.7%) | 750 (38.9%) |  |  |
| Always | 837 (63.0%) | 147 (52.1%) | 150 (61.2%) | 44 (60.3%) | 1178 (61.1%) |  |  |
| Chronic disease |  |  |  |  |  | 0.718 ^a^ | 0.869 |
| Yes | 131 (9.9%) | 31 (11.0%) | 25 (10.2%) | 9 (12.3%) | 196 (10.2%) |  |  |
| No | 1197 (90.1%) | 251 (89.0%) | 220 (89.8%) | 64 (87.7%) | 1732 (89.8) |  |  |
| Self-assessment of health status |  |  |  |  |  | 22.671 ^a^ | <0.001*** |
| Very Satisfied Good | 399 (30.0%) | 46 (16.3%) | 63 (25.7%) | 22 (30.1%) | 530 (27.5%) |  |  |
| At least satisfactory Fair/poor | 929 (66.5%) | 236 (83.7%) | 182 (74.3%) | 51 (69.9%) | 1398 (72.5%) |  |  |
| Working experience during COVID-19 epidemic |  |  |  |  |  | 16.307 ^a^ | 0.001** |
| No | 1063 (80.0%) | 246 (87.2%) | 208 (84.9%) | 68 (93.2%) | 1585 (82.2%) |  |  |
| Yes | 265 (20.0%) | 36 (12.8%) | 37 (15.1%) | 5 (6.8%) | 343 (17.8%) |  |  |
| Vaccine-related knowledge level |  |  |  |  |  | 11.994 ^a^ | 0.007** |
| Fail | 512 (38.6%) | 139 (49.3%) | 92 (37.6%) | 28 (38.4%) | 771 (40.0%) |  |  |
| Pass | 816 (61.4%) | 143 (50.7%) | 153 (62.4%) | 45 (61.6%) | 1157 (60.0%) |  |  |

*Notes.* *p < 0.05, **p < 0.01, ***p < 0.001.
